# Association of type 2 diabetes mellitus with lung cancer in patients with chronic obstructive pulmonary disease

**DOI:** 10.3389/fmed.2023.1118863

**Published:** 2023-04-27

**Authors:** Nam Eun Kim, Eun-Hwa Kang, Eunhee Ha, Ji-Young Lee, Jin Hwa Lee

**Affiliations:** ^1^Division of Pulmonary and Critical Care Medicine, Department of Internal Medicine, College of Medicine, Ewha Womans University, Seoul, Republic of Korea; ^2^Informatization Department, Ewha Womans University Medical Center, Seoul, Republic of Korea; ^3^Graduate Program in System Health Science and Engineering, Department of Environmental Medicine, College of Medicine, Ewha Medical Research Institute, Ewha Womans University, Seoul, Republic of Korea; ^4^Inflammation-Cancer Microenvironment Research Center, College of Medicine, Ewha Womans University, Seoul, Republic of Korea

**Keywords:** chronic obstructive pulmonary disease, diabetes mellitus, lung cancer, smoking, cohort study

## Abstract

**Background:**

Patients with chronic obstructive pulmonary disease (COPD) have an increased risk of developing lung cancer. Some studies have also suggested that diabetes mellitus (DM) may increase the risk of developing lung cancer. This study aimed to investigate whether type 2 DM (T2DM) is associated with an increased risk of lung cancer in patients with COPD.

**Materials and methods:**

We conducted a retrospective analysis on two cohorts: the National Health Insurance Service-National Sample Cohort (NHIS-NSC) of Korea and the Common Data Model (CDM) database of a university hospital. Among patients newly diagnosed with COPD in each cohort, those with a lung cancer diagnosis were included, and a control group was selected through propensity score matching. We used the Kaplan–Meier analysis and Cox proportional hazard models to compare lung cancer incidence between patients with COPD and T2DM and those without T2DM.

**Results:**

In the NHIS-NSC and CDM cohorts, we enrolled 3,474 and 858 patients with COPD, respectively. In both cohorts, T2DM was associated with an increased risk of lung cancer [NHIS-NSC: adjusted hazard ratio (aHR), 1.20; 95% confidence interval (CI), 1.02–1.41; and CDM: aHR, 1.45; 95% CI, 1.02–2.07). Furthermore, in the NHIS-NSC, among patients with COPD and T2DM, the risk of lung cancer was higher in current smokers than in never-smokers (aHR, 1.45; 95% CI, 1.09–1.91); in smokers with ≥30 pack-years than in never-smokers (aHR, 1.82; 95% CI, 1.49–2.25); and in rural residents than in metropolitan residents (aHR, 1.33; 95% CI, 1.06–1.68).

**Conclusion:**

Our findings suggest that patients with COPD and T2DM may have an increased risk of developing lung cancer compared to those without T2DM.

## 1. Introduction

Lung cancer is the second most commonly diagnosed cancer worldwide and the leading cause of cancer mortality, accounting for 18% of deaths in both men and women (1, 2). Chronic obstructive pulmonary disease (COPD) increases the risk of lung cancer due to shared etiologies, such as smoking, environmental pollution exposure, genetic predisposition, and inflammation pathways (3–5). Studies have also shown that COPD is an independent risk factor for lung cancer, with a six-fold higher risk regardless of smoking status (6, 7). However, COPD is a heterogeneous disease with various clinical phenotypes and comorbidities that can worsen its clinical outcomes and cause systemic inflammation (8). Type 2 diabetes mellitus (T2DM) is one of the most common comorbidities in patients with COPD. A cohort study of 2,164 patients with COPD found that diabetes mellitus (DM) increased mortality (9).

Diabetes mellitus (DM) is a systemic disorder characterized by hyperglycemia, inflammation, and oxidative stress that can affect organs such as the kidney, retina, and vascular system (10–12). DM may also influence lung cancer through mechanisms, such as hyperinsulinemia, hyperglycemia, or chronic inflammation, which have been associated with cancer progression (13, 14). However, the association between DM and lung cancer is inconclusive. While some studies did not find an increased risk of lung cancer in patients with DM (15, 16), other studies suggested a higher risk (17–19). Since T2DM is prevalent in patients with COPD and may increase the risk of developing lung cancer, this possible association requires further investigation.

To determine whether T2DM is associated with an increased risk of lung cancer in patients with COPD, we conducted a retrospective analysis on two cohorts: the National Health Insurance Service-National Sample Cohort (NHIS-NSC) of Korea and the Common Data Model (CDM) database of a university hospital.

## 2. Materials and methods

### 2.1. Study population

The NHIS in Korea maintains records of all covered inpatient and outpatient visits, procedures, and prescriptions. The NHIS-NSC is a nationwide, retrospective cohort from 2002 to 2019, consisting of data on a sample population of 1,137,896 patients selected randomly from the NHIS database and followed up until 2019.

The CDM database is generated by removing personal information from electronic health record data of 1,825,156 patients treated at Ewha Womans University Mokdong Hospital from 2001 to 2019. It includes information on diagnosis, laboratory examinations, medication, surgery, and other relevant medical data.

Our study included all patients aged 35–90 years from both cohorts. For the NHIS-NSC study patients, we limited the analysis to those who underwent a national health examination with a smoking history at least once during the study period.

We identified patients with COPD, T2DM, and lung cancer as primary or secondary diagnoses by searching for codes from the International Classification of Diseases, Tenth Revision (ICD-10). COPD was defined by the following criteria: patients who were (1) treated with ICD-10 codes for COPD (J43.x-J44.x, except J43.0), and (2) prescribed COPD medications at least twice a year between 1 January 2004, and 31 December 2018. The COPD medications included inhaled long-acting muscarinic antagonists, inhaled long-acting beta-2 agonists (LABAs), inhaled corticosteroids (ICSs), ICS plus LABA, inhaled short-acting muscarinic antagonists, and inhaled short-acting beta-2 agonists. For the CDM cohort data, the lung function definition of COPD was additionally applied. COPD was also defined when the ratio of post-bronchodilator forced expiratory volume in 1 s (FEV1)/forced volume vital capacity (FVC) was <0.7 at least twice between 2002 and 2018, along with the COPD ICD-10 diagnostic codes, even if a COPD medication prescription was not confirmed.

Patients with a history of COPD before 2004 or a history of lung cancer before being diagnosed with COPD were excluded from the study.

T2DM was defined by the following criteria: patients who were (1) treated with ICD-10 codes for T2DM (E11.x–E14.x) and (2) prescribed anti-diabetic medication (metformin [MET], sulfonylurea [SU], meglitinide, alpha-glucosidase inhibitor, MET plus SU, MET plus thiazolidinedione [TZD], MET plus dipeptidyl peptidase-4 inhibitor, or SU plus TZD) at least once a year.

Lung cancer was defined by the ICD-10 code C34.x.

Among the patients newly diagnosed with COPD during our study period, those diagnosed with lung cancer were included. To select a control group consisting of patients without lung cancer among the patients with COPD, 1:5 propensity score matching was performed on randomly selected patients by matching age, sex, and enrollment year.

This study was approved by the institutional review board (IRB) of Ewha Womans University Medical Center, Seoul, Republic of Korea (IRB number: SEUMC2021-11-007). The IRB waived the need to obtain informed consent considering the retrospective nature of the study. All methods were performed in accordance with the relevant guidelines and regulations laid down in the latest revision of the Declaration of Helsinki.

### 2.2. Statistical analysis

Descriptive statistics of the patients are expressed as means (standard deviation) or numbers (%). We used the *t*-test for continuous variables and chi-square test for categorical variables. We employed Kaplan–Meier analysis and the log-rank test to examine the statistical significance of the differences in lung cancer incidence between patients with COPD and T2DM and those without T2DM. Cox proportional hazard models were constructed to evaluate the effects of T2DM on the risk of lung cancer while adjusting for available confounders such as age, sex, smoking status, household income, residential area, body mass index (BMI), and the Charlson Comorbidity Index (CCI) (20). Hazard ratios (HRs) with 95% confidence intervals (CIs) were calculated for the risk of lung cancer. Moreover, we conducted subgroup analyses of patients with COPD and T2DM, stratified by age, sex, smoking status, household income, residential area, BMI, and CCI, which are strong confounders for lung cancer. We managed the large amount of data using SAS version 9.4 (SAS Institute, Cary, NC, USA) and conducted data analysis using R software version 4.0.3 (R Development Core Team, 2013). The significance level was set at *P* < 0.05.

## 3. Results

**FIGURE 1:**
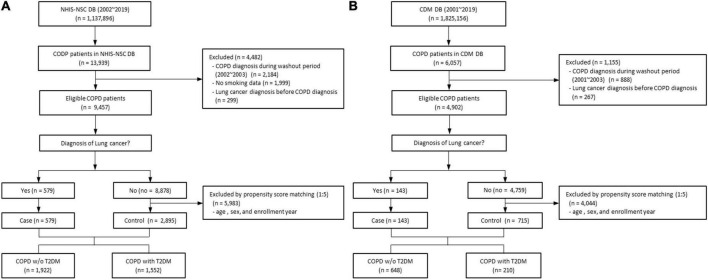
Flow chart of study design of **(A)** the NHIS-NSC and **(B)** the CDM cohort. CDM, common data model; DM, database; NHIS-NSC, national sample cohort of National Health Insurance Service; T2DM, type 2 diabetes mellitus; w/o, without.

Based on the NHIS-NSC database, we enrolled patients who were newly diagnosed with COPD and aged between 35 and 90 years from 2002 to 2018 (*n* = 13,939). We excluded patients who were diagnosed with COPD during a washout period from 2002 to 2003 (*n* = 2,184), those with missing values for smoking status or BMI (*n* = 1,999), and those who were diagnosed with lung cancer before or within one month after a COPD diagnosis (*n* = 299). Each patient was followed up until the development of lung cancer, death, or the end of 2019, whichever occurred first. Among the remaining patients, 579 with COPD were diagnosed with lung cancer in the NIHS-NSC database. We performed 1:5 propensity score matching to select a control group without lung cancer patients among eligible patients with COPD. In total, 3,474 patients were selected, consisting of 1,922 without T2DM and 1,552 with T2DM (Figure 1A).

From the CDM cohort, there were 6,057 patients, aged 35–90 years, who were newly diagnosed with COPD between 2001 and 2018. We excluded patients diagnosed with COPD during a washout period from 2001 to 2003 (*n* = 888) and those diagnosed with lung cancer before or within one month after a COPD diagnosis (*n* = 267). Each patient was followed up until the development of lung cancer, death, or the end of 2019, whichever occurred first. Finally, there were 143 patients with COPD diagnosed with lung cancer in the CDM cohort. We performed 1:5 propensity score matching to select a control group without lung cancer patients among eligible patients with COPD, using the same criteria as in the NHIS dataset. In total, 858 patients were selected, including 648 without T2DM and 210 with T2DM (Figure 1B).

Table 1 summarizes the baseline demographic characteristics of patients with COPD with and without T2DM in each of the two cohorts. In the NHIS-NSC, patients with COPD and T2DM had a higher mean BMI and a higher proportion of rural residents than those without T2DM. There was no significant difference in age, sex, smoking status, or smoking pack-years. In the CDM cohort, patients with COPD and T2DM had a higher mean BMI and white blood cell counts than those without T2DM. There were more cases of CCI values ≥1 in patients with COPD and T2DM. There was no significant difference in age, sex, or predicted FEV_1_, FVC, or FEV_1_/FVC ratio. Smoking data could not be compared due to the absence of information in the CDM cohort.

**TABLE 1:** Baseline characteristics of COPD patients with or without T2DM.

Characteristics	NHIS-NSC cohort			CDM cohort		
	**Without T2DM (*n* = 1,922)**	**With T2DM (*n* = 1,552)**	***P*** -value*	**Without T2DM (*n* = 648)**	**With T2DM (*n* = 210)**	***P*** -value*
Age (years)	78.2 ± 8.7	78.4 ± 8.5	0.513	71.1 ± 9.7	71.5 ± 8.1	0.648
35–60	148 (7.7)	104 (6.7)	0.526	97 (15.0)	22 (10.48)	0.261
60–70	456 (23.7)	370 (23.8)		157 (24.2)	54 (25.71)	
70–90	1,318 (68.6)	1,078 (69.5)		394 (60.8)	134 (63.81)	
Men	1,649 (85.8)	1,354 (87.2)	0.216	604 (93.2)	188 (89.5)	0.081
BMI (kg/m^2^)	22.6 ± 3.5	23.6 ± 3.7	0.001	22.6 ± 3.4	23.8 ± 3.8	<0.001
**CCI**						
0	579 (30.1)	472 (30.4)	0.854	142 (21.9)	23 (11.1)	<0.001
≥1	1,343 (69.8)	1,080 (69.6)		506 (78.1)	187 (89.0)	
**Smoking status**						
Never	762 (39.7)	612 (39.4)	0.988	–	–	–
Former	745 (38.8)	602 (38.8)		–	–	
Current	415 (21.6)	338 (21.8)		–	–	
Smoking (pack-years)	15.3 ± 23.5	16.1 ± 23.8	0.324	–	–	–
**Household income**						
<$ 2,000	730 (38.0)	543 (35.0)	0.177	–	–	–
2,000–5,000	548 (28.5)	472 (30.4)		–	–	
>$ 5,000	644 (33.5)	537 (34.6)		–	–	
**Residential area**						
Metropolitan	777 (40.4)	571 (36.8)	0.021	–	–	–
Urban	831 (43.2)	649 (41.8)		–	–	
Rural	314 (16.4)	332 (21.4)		–	–	
**Lung function**						
FEV_1_% predicted	–	–		70.4 ± 21.8	66.7 ± 22.4	0.089
<50	–	–		307 (47.4)	101 (48.1)	0.150
≥50	–	–		76 (11.7)	35 (16.7)	
Missing	–	–		265 (40.9)	74 (35.3)	
FVC% predicted				80.0 ± 17.2	74.8 ± 17.7	0.074
FEV_1_/FVC (%)				68.5 ± 21.6	65.4 ± 22.9	0.178
**Laboratory findings**						
WBC (×10^3^/mm^3^)	–	–	–	8.8 ± 3.8	9.9 ± 4.3	< 0.001
Hemoglobin (g/dl)	–	–	–	13.5 ± 2.0	13.1 ± 2.0	0.051
Platelets (×10^3^/mm^3^)	–	–	–	233.8 ± 83.2	233.2 ± 86.0	0.929
C-reactive protein (mg/dl)	–	–	–	1.3 ± 3.2	1.4 ± 2.8	0.853

Data are presented as number (%) or mean ± SD. BMI, body mass index; CCI, Charlson Comorbidity Index; CDM, common data model; FEV_1_, forced expiratory volume in one second; FVC, forced vita capacity; NHIS-NSC, national sample cohort of National Health Insurance Service; T2DM, type 2 diabetes mellitus; WBC, white blood cell.

*Two-sided χ^2^ test and *t*-test where appropriate.

**FIGURE 2:**
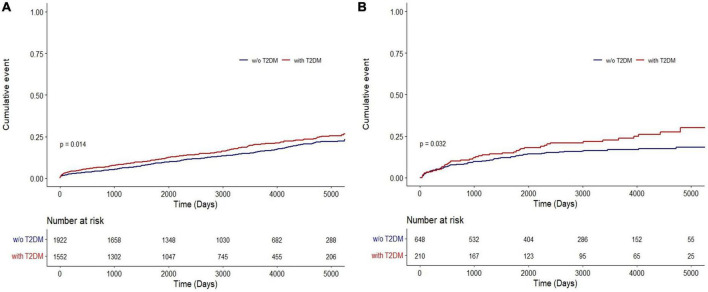
Cumulative incidence of lung cancer in COPD patients with and without T2DM in **(A)** the NHIS-NSC and **(B)** the CDM cohort. CDM, common data model; NHIS-NSC, national sample cohort of National Health Insurance Service; T2DM, type 2 diabetes mellitus; w/o, without.

Results of the Kaplan–Meier analysis showed a higher cumulative lung cancer incidence in patients with COPD and T2DM than in those without T2DM (NHIS-NSC: *P* = 0.014; CDM: *P* = 0.032) (Figure 2).

**FIGURE 3:**
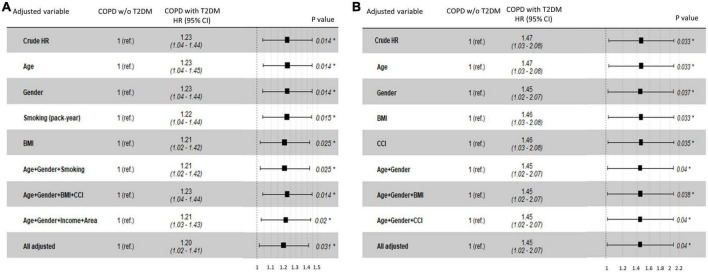
Hazard ratios for lung cancer risk in COPD patients with T2DM compared with those without T2DM in **(A)** the NHIS-NSC and **(B)** the CDM cohort. BMI, body mass index; CCI, Charlson Comorbidity Index; CDM, common data model; NHIS-NSC, national sample cohort of National Health Insurance Service; R. area, residential area; ref., reference; T2DM, type 2 diabetes mellitus; w/o, without.

In the NHIS-NSC, T2DM was associated with an increased risk of lung cancer (HR, 1.23; 95% CI, 1.04–1.44). When adjusting for age, sex, smoking pack-years, household income, residential area, BMI, and CCI, the risk of lung cancer was significantly higher in patients with COPD and T2DM than in those without T2DM (adjusted HR [aHR], 1.20; 95% CI, 1.02–1.41; Figure 3A). Among patients with COPD from the CDM cohort, T2DM was independently associated with an increased risk of lung cancer after adjusting for age, sex, BMI, and CCI (aHR, 1.45; 95% CI, 1.02–2.07; Figure 3B).

A subgroup analysis was performed in patients with COPD and T2DM to determine whether there was a difference in the risk of lung cancer according to age, sex, BMI, CCI, smoking pack-years, household income, and residential area (Table 2). In the NHIS-NSC, the risk of lung cancer was higher in current smokers than in never-smokers (aHR, 1.45; 95% CI, 1.09–1.91); in smokers with ≥30 pack-years than in never-smokers (aHR, 1.82; 95% CI, 1.49–2.25); and in rural residents than in metropolitan residents (aHR, 1.33; 95% CI, 1.06–1.68).

**TABLE 2:** Subgroup analysis: the risk of lung cancer in COPD patients with T2DM.

	COPD with T2DM in NHIS-NSC (*n* = 1,552)			COPD with T2DM in CDM (*n* = 210)		
	***N***	**Crude HR** **(95% CI)**	**Adjusted HR** **(95% CI)**	***N***	**Crude HR** **(95% CI)**	**Adjusted HR** **(95% CI)**
**Age**						
35–60	104	Ref	Ref	22	Ref	Ref
60–70	370	1.25 (0.84–1.87)	1.29 (0.87–1.93)	54	1.02 (0.66–1.57)	1.04 (0.67–1.63)
70–90	1,078	1.29 (0.90–1.86)	1.32 (0.91–1.91)	134	1.04 (0.67–1.62)	1.05 (0.68–1.64)
**Sex**						
Women	198	Ref	Ref	22	Ref	Ref
Men	1,374	1.07 (0.84–1.37)	1.01 (0.77–1.30)	188	0.85 (0.48–1.50)	0.85 (0.48–1.51)
**BMI, kg/m^2^**						
<23	741	Ref	Ref	66	Ref	Ref
≥23	811	1.09 (0.92–1.28)	1.18 (0.99–1.40)	87	1.92 (0.73–1.63)	1.07 (0.71–1.61)
**CCI**						
0	472	Ref	Ref	23	Ref	Ref
≥1	1,080	0.82 (0.69–0.98)	0.76 (0.64–0.91)	187	1.02 (0.67–1.57)	1.01 (0.65–1.58)
**Smoking status**						
Never	612	Ref	Ref	–	–	–
Former	609	1.12 (0.91–1.36)	0.90 (0.70–1.17)	–	–	–
Current	338	1.19 (1.55–2.36)*	1.45 (1.09–1.91)*	–	–	–
**Smoking (pack-years)**						
0	954	Ref	Ref	–	–	–
<30	308	1.25 (1.01–1.56)*	1.29 (1.03–1.61)*	–	–	–
≥30	290	1.76 (1.44–2.14)*	1.82 (1.49–2.25)*	–	–	–
**Household income**						
>$ 5,000	543	Ref	Ref	–	–	–
$ 2,000–5,000	472	1.10 (0.90–1.35)	1.11 (0.90–1.37)	–	–	–
<$ 2,000	537	1.03 (0.84–1.26)	1.04 (0.85–1.27)	–	–	–
**Residential area**						
Metropolitan	571	Ref	Ref	–	–	–
Urban	649	1.19 (0.99–1.44)	1.18 (0.98–1.43)	–	–	–
Rural	332	1.31 (1.04–1.65)*	1.33 (1.06–1.68)*	–	–	–

BMI, body mass index; CCI, Charlson Comorbidity Index; CDM, common data model; NHIS-NSC, national sample cohort of National Health Insurance Service; ref, reference; T2DM, type 2 diabetes mellitus.

*P < 0.05.

## 4. Discussion

In this study, through two independent cohort analyses, we found that patients with COPD and T2DM had a higher risk of developing lung cancer than those without T2DM, even after adjusting for possible confounding factors. Based on a subgroup analysis of patients with COPD and T2DM, the risk of lung cancer was increased among smokers, which is commonly observed in lung cancer prevalence (1). Additionally, in patients with COPD and T2DM, the risk of lung cancer was higher in rural residents than in metropolitan residents. These findings are consistent with those of a study by Jenkins et al. that reported increased lung cancer incidence and mortality rates in rural areas (21). In rural areas, people are less likely to consult a doctor due to low accessibility to hospitals, and it is difficult to participate in smoking cessation and health programs, which can increase the risk of cancer diagnosis (22, 23).

The hypothesis for our study was that T2DM, a well-known comorbidity of COPD, not only impacts the clinical outcomes of COPD but also increases the risk of lung cancer in patients with COPD. Various studies have investigated the possibility that diabetic microangiopathy can affect the lungs, leading to histological changes, functional abnormalities, and chronic inflammation at the molecular level (10, 24, 25). Recent case-control studies have also demonstrated that T2DM significantly decreases pulmonary function compared to healthy controls (26, 27). An experimental model has demonstrated that hyperglycemia can increase levels of inflammatory cytokines, including interleukin-6 and tumor necrosis factor-alpha, which play a crucial role in epithelial-to-mesenchymal transition and tumorigenesis (28). In our CDM cohort, patients with COPD and T2DM had higher mean white blood cell counts and, although not statistically significant, lower mean hemoglobin and higher mean C-reactive protein levels than those in patients without T2DM. These findings may indicate that T2DM increases systemic inflammation in patients with COPD.

Several retrospective cohort studies using the National Health Insurance (NHI) database in Taiwan have reported associations between diabetes, lung cancer, and COPD. Two studies, also using the NHI database, reported that diabetes increased the risk of lung cancer (18, 19). Tseng reported that age, sex, and COPD were positively associated with lung cancer among patients with DM (19). However, Shen et al. concluded that T2DM could have a protective effect against lung cancer in patients with COPD (29), which contradicts our findings. They speculated that the anti-inflammatory effect of anti-diabetic drugs might prevent lung cancer. Although many studies have reported that metformin, one of the most widely prescribed anti-diabetic drugs, reduces the risk of cancer (30, 31), a large observational study in the United Kingdom showed that metformin use was not associated with a decreased risk of lung cancer (32). Additionally, from our perspective, the chronic inflammation in T2DM, along with the chronic inflammation in COPD, is more likely to increase the risk of lung cancer.

Another important point is that while Shen et al. analyzed only the NHI data of Taiwan, we obtained the same results by analyzing the NHIS-NSC of Korea as well as the CDM cohort data of a university hospital. These two cohorts had complementary advantages. Although the NHIS-NSC is representative of the country, it has the disadvantage that the diagnosis of each disease requires manipulation of the data. In comparison, since the CDM cohort included lung function data and diagnosis by a relatively small number of respiratory specialists, the accuracy of the COPD diagnosis was high. While smoking history, the most important risk factor for lung cancer, is not available in the NHI database of Taiwan, the NHIS-NSC data included smoking history obtained during national health checkups.

Type 2 diabetes mellitus is a common comorbidity in patients with COPD and may increase the risk of lung cancer. COPD can activate inflammation pathways that increase oxidative stress, leading to DNA damage, which is a known trigger for lung cancer (33). Some studies have suggested that COPD may also induce T2DM, increase the risk for metabolic syndrome, and cause insulin resistance (24, 34). Although the causes and effects of the association between DM and COPD are unclear, they may influence the development of lung cancer.

Tobacco is a potent carcinogen, and its harmful effects, when smoked or exposed to nearby individuals intermittently, are well-established. In contrast, although the impact of T2DM on tumorigenesis may be less significant, persistent and frequent exposure to hyperglycemia can contribute to the development of cancer (35). Our study found that, after adjusting for smoking, a potential confounding factor in clinical settings, the HR of DM on COPD remained significant. Furthermore, results our subgroup analysis of patients with COPD and T2DM showed that current smokers with T2DM had a higher risk of lung cancer, suggesting a potential additive effect on the development of cancer. Finally, the higher risk of lung cancer in rural areas may be not only due to poor participation in smoking cessation programs but also inevitable glucose control for patients with T2DM due to limited access to medical resources.

This study has several strengths. First, the accuracy of the study was improved by using two independent cohorts. The NHIS-NSC dataset is a large, representative data source based on the national population, and it includes risk factors for lung cancer such as smoking status, household income, residential area, and BMI. In the CDM cohort, a spirometry-based COPD definition was additionally applied, and detailed medication information based on hospital data was available when selecting study patients. Diagnoses and prescriptions were confirmed by pulmonologists and endocrinologists, ensuring the high reliability of the diagnosis. The long follow-up period is another strength of this study.

However, our study has some limitations. In the CDM data, there is a lack of information about smoking, which is a major risk factor for lung cancer. Smoking data were recorded as text files in the electronic medical forms and could not be extracted. Nevertheless, similar to other studies representative of Korea (36), 92% of patients with COPD in this study were men, and given the high smoking rate among men in Korea, it is very likely that most were current or former smokers. Moreover, the results were very similar to those of the NHIS-NSC, which was analyzed using smoking status and pack-years. Second, although this study investigated a large dataset of patients with COPD, caution is needed in generalizing its results due to the limitations of a retrospective epidemiologic study. Third, the type of treatment for COPD and T2DM and its administration period, the degree of symptom and glycemic control, and the duration of T2DM may affect lung cancer incidence. However, analysis of these factors was not possible based on the current dataset.

Patients with COPD and T2DM have an increased risk of developing lung cancer compared with those without T2DM. Furthermore, among patients with both COPD and T2DM, the risk of lung cancer was found to be higher in current smokers and in smokers with ≥30 pack-years than in never-smokers, and in rural residents than in metropolitan residents. Although further studies are necessary, it is prudent to carefully consider the possibility of lung cancer during the follow-up of patients with COPD and T2DM.

## Data availability statement

The NHIS-NSC and CDM data may be provided through a remote virtualization room when an application is made to the National Health Insurance Service (https://nhiss.nhis.or.kr/bd/ab/bdaba001cv.do) and the Ewha Womans University Medical Center (http://part.eumc.ac.kr/dept/RDPC/index.do), respectively, and is approved. However, the use of the data is only available in Republic of Korea.

## Ethics statement

The studies involving human participants were reviewed and approved by the Institutional review board (IRB) of Ewha Womans University Medical Center, Seoul, Republic of Korea (IRB number: SEUMC2021-11-007) and the IRB waived the need to obtain informed consent considering the retrospective nature of the study. Written informed consent for participation was not required for this study in accordance with the national legislation and the institutional requirements.

## Author contributions

JL and JYL were the guarantors, took responsibility for the content of the manuscript, including the data and analysis, and were responsible for the acquisition of data and study supervision. EHK and JYL analyzed the data and created the figures. NK and EHK wrote the first draft of the manuscript. All authors contributed substantially to the study concept and design, interpreted the analysis results, critically revised the manuscript, provided supervision for the final manuscript, and approved the final draft of the manuscript.
